# Machine learning-selected inflammation biomarkers for stable coronary artery disease with intermediate coronary lesions: potential for long-term prognosis in a multicenter cohort study

**DOI:** 10.3389/fphys.2026.1688153

**Published:** 2026-02-17

**Authors:** Qiong Xu, Shoupeng Duan, Shuo Liu, Siyang Li, Zongchao Zuo, Jiajun Zhu, Jun Wang

**Affiliations:** 1 Department of Cardiology, The First Affiliated Hospital of Bengbu Medical University, Bengbu, Anhui, China; 2 Department of Cardiology, Renmin Hospital of Wuhan University, Wuhan, Hubei, China; 3 First Clinical College, Anhui Medical University, Hefei, Anhui, China; 4 Department of Cardiology, Xiangyang Central Hospital, Affiliated Hospital of Hubei University of Arts and Science, Xiangyang, Hubei, China; 5 Department of Cardiology, the First Affiliated Hospital of Xinjiang Medical University, Urumchi, China; 6 Joint Research Center for Regional Diseases of IHM, Bengbu Medical University, Bengbu, Anhui, China; 7 Joint Research Center for Regional Diseases of IHM, The First Affiliated Hospital of Bengbu Medical University, Bengbu, Anhui, China

**Keywords:** inflammation biomarkers, intermediate coronary lesions, machine l earning, prediction nomogram, stable coronary artery disease

## Abstract

**Background:**

Stable coronary artery disease (SCAD) generally exhibits prolonged periods of stability. However, this condition can unpredictably progress into an unstable state, representing a complex pathological process involving multiple contributing factors. Thus, we aimed to utilize machine-learning techniques to identify predictive features from electronic health record (EHR) data for forecasting the long-term prognosis of patients with SCAD and intermediate coronary lesions.

**Methods:**

Patients were divided into a training cohort (n = 403) and an external validation cohort (n = 247) according to their hospital of origin during the period from January 2018 to December 2020. Predictive features were determined using LASSO regression analysis and boruta algorithm, followed by multivariate Cox regression analysis for model construction.

**Results:**

The developed predictive model comprised four clinical variables: platelet-to-lymphocyte ratio, diabetes mellitus, lipoprotein(a), and mean platelet width. The area under the curve for predicting major adverse cardiovascular events (MACEs) within 2-, 3- and 4-year in the development cohort was 0.692 (95%CI:0.59-0.793), 0.709 (95%CI:0.625-0.792) and 0.743 (95%CI:0.672-0.813), respectively, while that in the external validation cohort was 0.658 (95%CI 0.542-0.773), 0.681 (95%CI:0.579-0.782) and 0.723 (95%CI: 0.635-0.811), respectively. Additionally, the developed predictive model was calibrated by analyzing the correlation between expected and observed MACEs in the development and external validation cohorts. Lastly, the clinical value of the developed predictive model was confirmed via decision curve analysis.

**Conclusion:**

Our validated nomogram was based on inflammation biomarkers and EHR data, demonstrating moderate discriminative ability to detect individuals at high risk of poor outcome among patients with SCAD and angiographically intermediate coronary stenosis.

## Introduction

Stable coronary artery disease (SCAD) is a complex pathological condition characterized by the accumulation of atherosclerotic plaques within the epicardial arteries, regardless of the degree of arterial obstruction caused by the plaques (Boden et al., 2007). SCAD entails a dynamic pathogenesis process influenced by numerous factors (Knuuti et al., 2025). Although SCAD typically presents with extended periods of stability, it can also undergo unpredictable destabilization (Knuuti et al., 2025). Moreover, SCAD is a chronically progressive condition with substantial clinical relevance, even during seemingly asymptomatic periods (Knuuti et al., 2025; Boden et al., 2023; Tang et al., 2023; Zhou B. et al., 2024). The clinical prognosis of some patients with SCAD and angiographically intermediate coronary stenosis may be adversely affected by treatment hesitancy, particularly when the degree of narrowing in such stenoses does not meet the severity threshold for immediate intervention (Wang et al., 2024a). Additionally, several complications may persist during and after percutaneous coronary intervention (PCI), including challenges in completely mitigating risks such as coronary restenosis and in-stent thrombosis. Moreover, accumulating evidence indicates that compared to conservative drug therapy, stent implantation does not provide supplementary advantages to patients with SCAD and angiographically intermediate coronary stenosis (Knuuti et al., 2025; Boden et al., 2007). Hence, future research should focus on enhancing the accuracy in identifying the subset of patients with SCAD who could benefit from PCI or pharmacological therapy, particularly in those with angiographically intermediate coronary stenosis.

Previous studies have shown that prediction nomograms, which integrates multi-modal data (including inflammatory biomarkers, clinical phenotype, demographic characteristics and comorbidities), reliably predicts both in-hospital and long-term prognoses across different subtypes of coronary heart disease (CHD) (Tang et al., 2023; Zhou B. et al., 2024; Wang et al., 2024a). However, limited studies have demonstrated the potential effectiveness of integrating multimodal data within a predictive model for providing personalized risk stratification of patients with SCAD and angiographically intermediate coronary stenosis. In our study, we employed diverse machine learning-driven inflammation biomarkers along with relevant clinical data extracted from patients’ electronic health records (EHRs). Based on these combined data, we aimed to construct a long-term predictive model to identify crucial prognostic factors for patients with SCAD and intermediate coronary stenosis, thereby augmenting disease management in this specific patient population.

## Methods

### Study design and patient selection

A total of 650 patients with SCAD and intermediate coronary stenosis who were hospitalized in First Affiliated Hospital of Xinjiang Medical University and Renmin Hospital of Wuhan University between January 2018 and December 2020 were consecutively screened. During their hospitalization, all enrolled patients underwent coronary angiography and not PCI to accurately assess their coronary vessels. Further, data from different hospital sources were used to develop a model development cohort and an independent validation cohort. Consequently, all patients were divided into a development cohort (n = 403; datasets from Renmin Hospital of Wuhan University) and independent validation cohort (n = 247; datasets from the First Affiliated Hospital of Xinjiang Medical University) according to their date of hospitalization. Patients who satisfied the subsequent criteria were not included in the study: severe infections, severe renal insufficiency (estimated glomerular filtration rate, [eGFR] < 30 mL/min), immune system diseases, life-limiting diseases other than coronary heart disease, and tumor-related diseases.

### Biochemical tests

A comprehensive biochemical tests was conducted for all patients prior to their scheduled coronary angiography. White blood cell count, neutrophil count, lymphocyte count, platelet count, monocyte count, neutrophil-to-lymphocyte ratio (NLR), platelet-to-lymphocyte ratio (PLR), and high-sensitivity C-reactive protein serve as key inflammatory biomarkers (Tang et al., 2023; Li et al., 2018).

### Coronary angiography

All patients underwent coronary angiography (Philips FD20 digital subtraction angiography machine, the Netherlands), and two independent interventionists with clinical experience estimated the degree of stenosis at each coronary lesion. In case of disagreements, a third interventionist was approached to reach a consensus. Intermediate coronary stenosis was defined as 50%–70% stenosis.

### Follow-up

Clinical follow-up of the study patients was conducted either in the clinic or via telephone. The primary objective of our research was to evaluate the occurrence of major adverse cardiac events (MACEs), including cardiac mortality, revascularization, and myocardial infarction (MI), as the composite endpoint.

### Feature selection and model construction

Our study employed a two-step statistical strategy designed to identify and validate predictors of MACEs in patients with stable coronary artery disease and intermediate coronary stenosis. We utilized LASSO regression analysis and Boruta algorithm to identify relevant features linked to MACEs in patients diagnosed with SCAD and intermediate coronary stenosis. Following this, multivariate Cox regression analysis was performed to assess the independent predictive ability of these variables for MACEs at 2, 3 and 4 years. This comprehensive approach ensures robust feature selection through the complementary strengths of both algorithms - LASSO regression provides automated variable selection with L1 regularization to prevent overfitting, while the Boruta algorithm offers robust feature importance assessment based on random forest methodology. The subsequent multivariate Cox regression analysis allows for evaluation of independent prognostic factors while accounting for potential confounders, with temporal assessment at multiple clinically relevant time points providing insights into both short-term and intermediate-term risk stratification.

The nomogram performance underwent rigorous multi-level validation to ascertain its reliability and practicality (Balachandran et al., 2015). First, receiver operating characteristic (ROC) curves and time-area under the curve (AUC) were generated to comprehensively assess the accuracy and discrimination ability of the nomogram in predicting MACE risk. To illustrate the correlation between the anticipated likelihood of MACEs using the nomogram and the real observed results in patients, a calibration graph was generated. This step facilitated nomogram calibration across different levels of predicted probability. To assess the overall advantage of the nomogram at different threshold probabilities, a decision curve analysis (DCA) was performed. Finally, scores were assigned to each patient based on their respective nomogram predictions in the development and independent validation cohorts, followed by categorizing the patients into high- and low-risk groups according to median values. The Kaplan–Meier survival curves were then employed to analyze the potential predictive validity of the two risk groups.

### Statistical analysis

All statistical analyses were conducted utilizing R 4.2.2 software. A descriptive statistical analysis of the baseline clinical data of the enrolled patients was initially performed. Furthermore, categorical variables were expressed as frequencies and proportions, while continuous variable distributions were presented as medians and interquartile ranges or means and standard deviations. The Fisher’s exact or χ^2^ tests were used to compare between the distribution of categorical variables in the development and independent validation groups. Furthermore, the t-test, Mann–Whitney U test, or Kruskal–Wallis test were utilized to analyze continuous variables considering their adherence to normal distribution and homogeneity. The significance level for all comparisons was set at P < 0.05.

## Results

### Patient characteristics

We retrospectively screened 650 patients with SCAD and angiographically intermediate coronary stenosis. All patients were divided into a development cohort (n = 403; datasets from January 2018 and December 2020) and validation cohort (n = 247; datasets from January 2018 and December 2020) according to their date of hospitalization. The average age of all 650 patients was 64 years, among which 409 (62.9%) were males. The baseline characteristics and results of the two cohorts were balanced (Table 1). During the median follow-up of 48 months, the overall incidence of MACEs was 16.0%, with similar rates of MACEs observed across the two cohorts.

**TABLE 1:** Baseline characteristics of patients in the development and validation cohorts.

Characteristic of outcome	All cohort (n = 650)	Development cohort (n = 403)	Validation cohort (n = 247)	P
Male, n (%)	241 (37.08%)	157 (38.96%)	84 (34.01%)	0.236
Age (years)	64.00 [58.00; 71.00]	64.00 [58.00; 70.00]	64.00 [58.00; 71.00]	0.938
Hypertension (%)	427 (65.69%)	272 (67.49%)	155 (62.75%)	0.250
Diabetes mellitus (%)	148 (22.77%)	87 (21.59%)	61 (24.70%)	0.412
Current smoking (%)	232 (35.69%)	149 (36.97%)	83 (33.60%)	0.432
Current alcohol consumption (%)	150 (23.08%)	94 (23.33%)	56 (22.67%)	0.924
Family history of hypertension (%)	90 (13.85%)	50 (12.41%)	40 (16.19%)	0.215
Family history of CAD (%)	71 (10.92%)	41 (10.17%)	30 (12.15%)	0.514
Family history of diabetes (%)	21 (3.23%)	11 (2.73%)	10 (4.05%)	0.487
History of myocardial infarction (%)	54 (8.31%)	30 (7.44%)	24 (9.72%)	0.383
Previous PCI (%)	142 (21.85%)	82 (20.35%)	60 (24.29%)	0.279
Neutrophil count (×109/L)	3.65 [2.89; 4.67]	3.72 [2.88; 4.63]	3.61 [2.94; 4.69]	0.813
Monocyte count (×109/L)	1.65 [1.26; 2.04]	1.63 [1.27; 2.04]	1.66 [1.25; 2.04]	0.950
NLR	2.23 [1.69; 3.04]	2.22 [1.68; 3.08]	2.23 [1.70; 3.01]	0.991
Platelet count (×109/L)	202.00 [167.00; 242.75]	204.00 [169.00; 244.50]	198.00 [165.00; 239.50]	0.401
PLR	124.16 [94.72; 159.51]	126.38 [96.02; 158.92]	121.88 [92.51; 160.05]	0.618
Mean platelet width (fL)	12.10 [10.90; 13.90]	12.10 [10.90; 13.85]	12.30 [10.90; 14.20]	0.387
Hs-CRP (mg/L)	2.00 [1.18; 3.33]	2.00 [1.25; 3.38]	2.00 [1.02; 3.12]	0.642
eGFR, mL/(min1.73 m^2^)	90.76 [79.86; 99.12]	91.00 [80.68; 99.57]	90.33 [77.34; 97.73]	0.176
Uric acid (mmol/L)	356.50 [291.25; 429.75]	354.00 [293.00; 433.00]	364.00 [290.00; 427.00]	0.947
Fasting plasma glucose (mmol/L)	5.90 [5.03; 7.55]	5.95 [5.04; 7.56]	5.82 [5.02; 7.54]	0.806
TG (mmol/L)	1.49 [1.04; 2.20]	1.48 [1.07; 2.24]	1.53 [1.02; 2.12]	0.704
TC (mmol/L)	4.02 [3.29; 4.80]	4.08 [3.33; 4.78]	3.91 [3.26; 4.88]	0.358
HDL-C (mmol/L)	1.08 [0.91; 1.31]	1.07 [0.91; 1.30]	1.10 [0.91; 1.31]	0.601
LDL-C (mmol/L)	2.26 [1.70; 2.97]	2.31 [1.71; 2.97]	2.16 [1.66; 3.00]	0.264
Lipoprotein a (g/L)	155.50 [73.00; 376.25]	157.00 [73.00; 391.00]	153.00 [72.00; 334.50]	0.596
TBil (μmol/L)	11.40 [8.80; 14.40]	11.20 [8.75; 14.60]	11.80 [8.90; 14.30]	0.605
DBil (μmol/L)	3.70 [2.80; 4.80]	3.70 [2.80; 4.80]	3.80 [2.85; 4.70]	0.514
Fibrinogen (g/L)	2.91 [2.47; 3.44]	2.93 [2.49; 3.49]	2.88 [2.47; 3.40]	0.389
AAOD (mm)	33.00 [31.00; 36.00]	33.00 [31.00; 36.00]	33.00 [31.00; 35.00]	0.412
MPAD (mm)	21.00 [20.00; 23.00]	21.00 [20.00; 22.00]	21.00 [20.00; 23.00]	0.532
LAD (mm)	36.00 [33.00; 39.00]	36.00 [33.00; 39.00]	36.00 [33.00; 38.00]	0.188
LVDD (mm)	45.00 [42.00; 47.00]	45.00 [42.00; 47.00]	44.00 [42.00; 47.00]	0.249
RAD (mm)	35.00 [33.00; 37.00]	35.00 [33.00; 37.00]	35.00 [33.00; 37.00]	0.196
RVD (mm)	21.00 [20.00; 22.00]	21.00 [20.00; 22.00]	21.00 [19.00; 22.00]	0.192
IVSD (mm)	10.00 [9.00; 10.00]	10.00 [9.00; 10.00]	10.00 [9.00; 11.00]	0.457
LVPWD (mm)	10.00 [9.00; 10.00]	10.00 [9.00; 10.00]	10.00 [9.00; 10.00]	0.341
LVEF (%)	60.00 [58.00; 60.00]	60.00 [59.50; 60.00]	60.00 [58.00; 60.00]	0.238
AVSV (cm/s)	126.00 [113.00; 141.00]	125.00 [113.00; 142.00]	126.00 [113.00; 138.00]	0.590
PVSV (cm/s)	88.00 [80.00; 99.00]	88.00 [79.00; 99.00]	88.00 [80.00; 97.50]	0.512
MVE (cm/s)	64.00 [55.00; 75.00]	64.00 [54.00; 74.00]	65.00 [55.00; 77.50]	0.240
MACEs (%)	104 (16.00%)	61 (15.14%)	43 (17.41%)	0.511
Cardiac mortality (%)	36 (5.54%)	23 (5.71%)	13 (5.26%)	0.949
Revascularization (%)	50 (7.69%)	29 (7.20%)	21 (8.50%)	0.649
Myocardial infarction (%)	20 (3.08%)	11 (2.73%)	9 (3.64%)	0.674

^a^ Values are given as n (%), mean (SD), or median (IQR).

CAD, coronary artery disease; PCI, percutaneous coronary intervention; NLR, neutrophil-to-lymphocyte ratio; PLR, platelet -to-lymphocyte ratio; hs-CRP, high-sensitivity C-reactive protein; eGFR, estimated glomerular filtration rate; TG, triglycerides; TC, total cholesterol; HDL-C, high-density lipoprotein cholesterol; LDL-C, low-density lipoprotein cholesterol; TBil, total bilirubin; DBil, Direct Bilirubin; AAOD, ascending aorta diameter; MPAD, main pulmonary artery diameter; LAD, left atrium diameter; LVDD, left ventricular end-diastolic diameter; RAD, right atrium diameter; RVD, right ventricular diameter; IVSD, interventricular septal thickness at diastole; LVPWD, left ventricular posterior wall in diastole; LVEF, left ventricular ejection fraction; AVSV, aortic valve systolic flow velocity; PVSV, pulmonary valve systolic flow velocity; MVE, mitral peak velocity of early filling; MACEs, major adverse cardiovascular events.

### Construction of the nomogram

**FIGURE 1:**
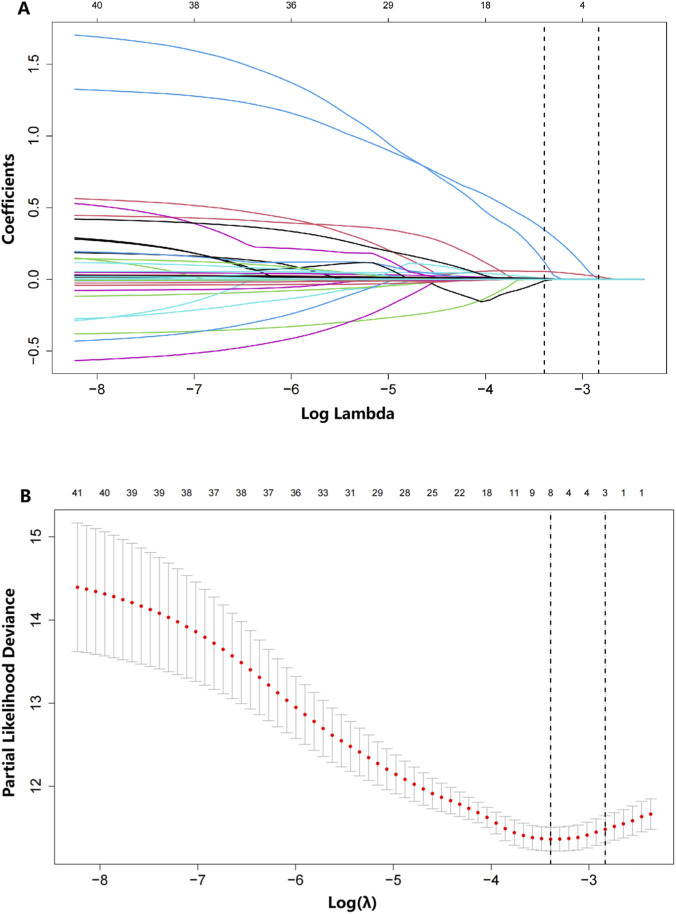
Prediction of long-term prognosis using 41 variables via the coefficient trend of the LASSO model **(A)**. Determination of the cross-validation error curve utilizing the tuning parameter (lambda, λ). According to the 10-fold cross-validation in the LASSO model, seven optimal nonzero coefficients are selected from a pool of 41 features based on their minimum mean cross-validation error **(B)**.

**FIGURE 2:**
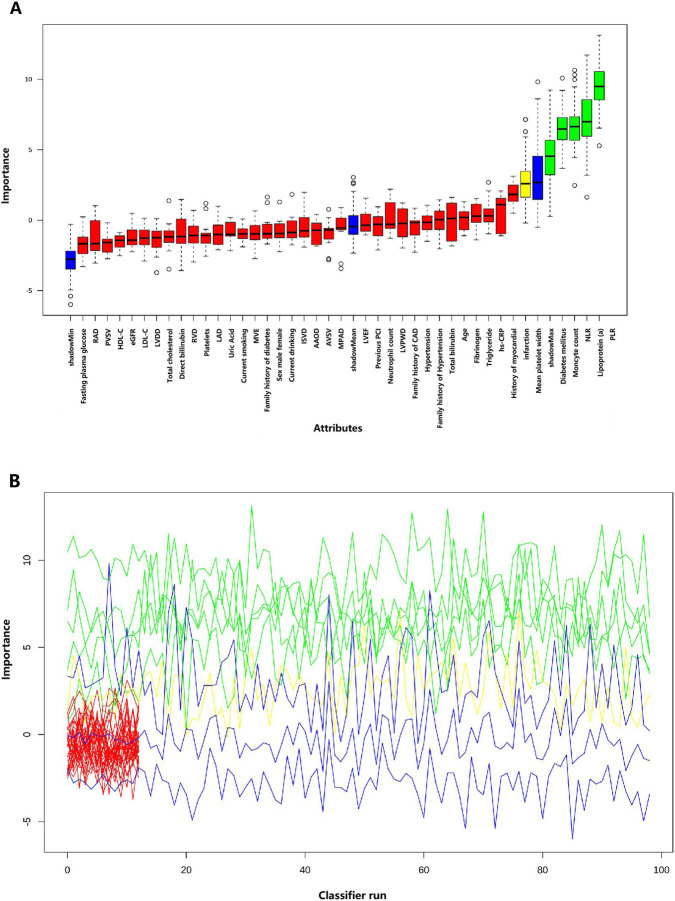
The Boruta algorithm was applied to identify potential predictors of patients. **(A)** The screening results of 41 clinical variables, with feature importance assessed using a random forest model. **(B)** The evolution of feature importance over 100 iterations of the Boruta algorithm.

LASSO regression screening was employed to identify the predictive features from 40 variables in the development cohort. Potential predictors of MACEs were identified through the detection of seven variables, including diabetes mellitus, history of myocardial infarction, monocyte count,NLR, PLR, mean platelet width, lipoprotein (a) and main pulmonary artery diameter (Figures 1A,B). The Boruta algorithm displayed in this visualization underscores the multifactorial nature of risk assessment and illustrates the potential value of machine learning in pinpointing key predictors from a wide range of clinical parameters (Figures 2A,B). The following variables were identified as significant predictors: diabetes mellitus, monocyte count, NLR, PLR, mean platelet width and lipoprotein (a). Through this dual approach, the following variables were identified as the most relevant predictors: diabetes mellitus, monocyte count, NLR, PLR, mean platelet width and lipoprotein(a). These variables were selected based on their consistent importance across both methods, ensuring a robust and reliable feature set for subsequent modeling and analysis.

**FIGURE 3:**
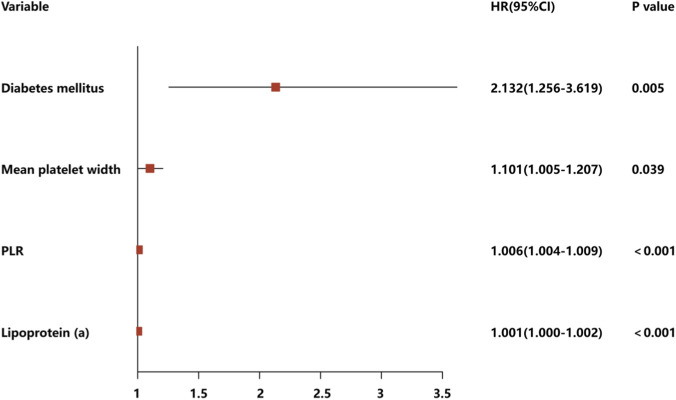
Multivariate Cox regression analysis of the development cohort to identify independent prognostic variables, along with forest plots to display the hazard ratios (HRs).

**FIGURE 4:**
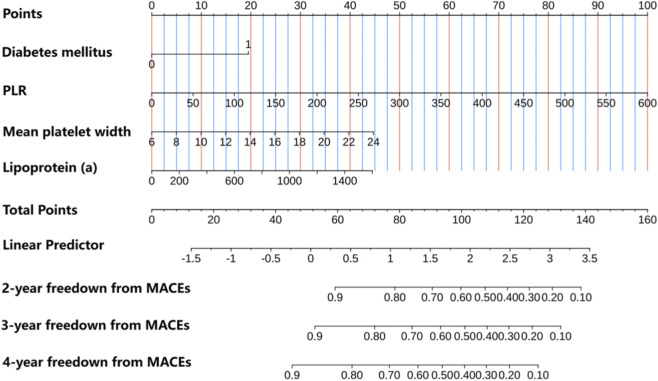
Development of a predictive nomogram via multivariate Cox regression analysis.

Diabetes mellitus (HR: 2.132, 95% CI: 1.256–3.619, P = 0.005), mean platelet width (HR: 1.101, 95% CI: 1.005–1.207, P = 0.039), PLR (HR: 1.006, 95% CI: 1.004–1.009, P < 0.001) and lipoprotein(a) (HR: 1.001, 95% CI: 1–1.002, P < 0.001), were determined as independent factors for MACEs through multivariate Cox regression analysis (Figure 3). Consequently, these four distinct variables were utilized to develop a prognostic nomogram capable of estimating the risk of MACEs over a period of 2, 3 and 4 years in patients diagnosed with SCAD and exhibiting coronary stenosis at an intermediate level (Figure 4).

### Performance of the nomogram

**FIGURE 5:**
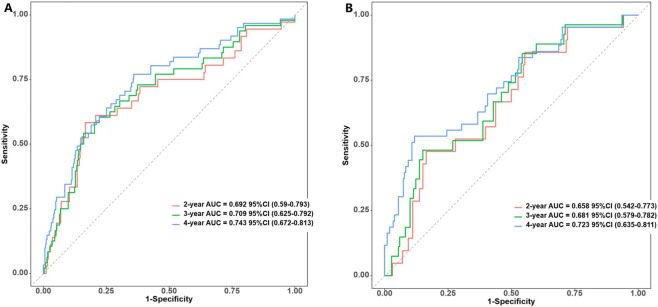
Receiver operating characteristic (ROC) curve analysis of the MACEs prediction accuracy of the nomogram in the development **(A)** and validation cohorts **(B)**.

We conducted an analysis to assess the effectiveness of the predictive nomogram in identifying patients with angiographically intermediate coronary stenosis who are at a heightened risk for MACEs. Time-dependent area under the curve (time-AUC) analyses demonstrated moderate predictive accuracy with acceptable model stability across datasets (Supplementary Figures S1A, S1B). In this evaluation, Harrell’s C-indexes and area under the curve (AUC) values were estimated. The C-indexes for discriminating low- and high-risk patients in the developmental and validation cohorts were 0.718 (95%CI: 0.651-0.785) and 0.697 (95%CI:0.619-0.775), respectively. Subsequently, the AUC values of the ROC for the predictive ability of the nomogram in forecasting MACE risk at 2-, 3- and 4-year intervals were 0.692 (95%CI:0.59-0.793), 0.709 (95%CI:0.625-0.792) and 0.743 (95%CI:0.672-0.813), respectively, in the development cohort. In the case of the independent validation cohort, the corresponding AUC values were 0.658 (95%CI 0.542-0.773), 0.681 (95%CI:0.579-0.782) and 0.723 (95%CI: 0.635-0.811) at the 2-, 3-, 4-year intervals, respectively (Figures 5A,B).

**FIGURE 6:**
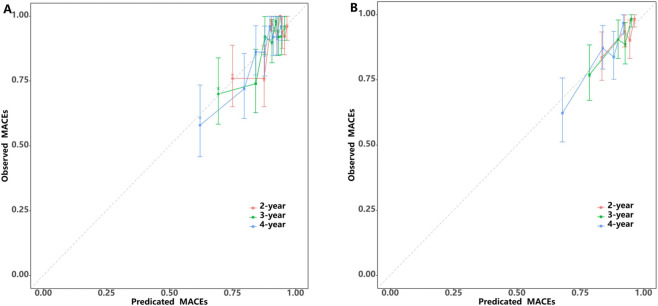
Calibration curves of the MACE-free survival predictors in the development **(A)** and validation cohorts **(B)**.

**FIGURE 7:**
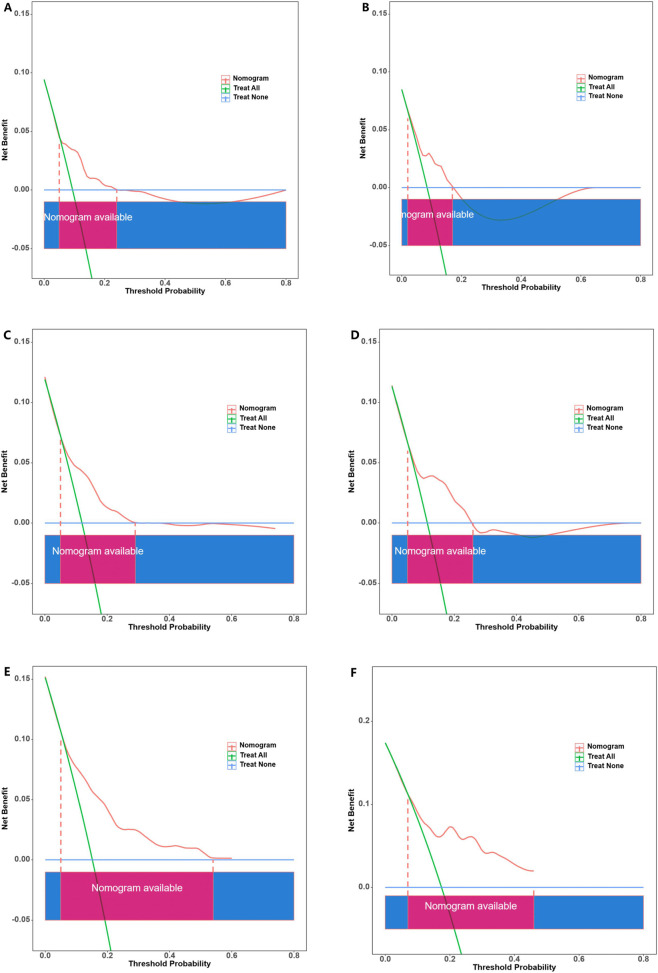
Decision curve analysis (DCA) for predicting MACE-free survival at 2 years **(A,D)** 3 years **(B,E)** and 4 years **(C,F)** in the development and validation cohorts, respectively.

Additionally, calibration plots were utilized to compare the predicted risk probabilities of the nomogram and observed outcomes for MACEs, aiming to discern MACE risk among patients having SCAD with intermediate coronary stenosis. The calibration plot analysis demonstrated that the nomogram’s predicted probabilities in both the development and validation cohorts aligned well with the actual risks of MACEs observed over a period of 2–4 years (Figures 6A,B). After assessing the nomogram’s performance, DCA was conducted to further validate the predictive efficacy of the nomogram. In the development cohort, the DCA findings revealed threshold probabilities ranging from 5% to 25% for predicting the likelihood of MACEs over a period of 2-year, from 3% to 30% over a period of 3-years and from 3% to 56% over a period of 4-years (Figures 7A–C). In the case of the independent validation, the DCA analysis demonstrated that the threshold probabilities for forecasting MACE likelihood over 2, 3 and 4 years were 5%–18%, 5%–26% and 5%–45%, respectively (Figures 7D–F). Furthermore, the DCA analysis established that the overall nomogram benefit was more advantageous than that of the five individual univariate models (diabetes mellitus, mean platelet width, PLR and lipoprotein(a)) in the development and independent validation cohorts (Supplementary Figures S2A–S2F). Moreover, the study patients in the development and independent validation cohorts were successfully stratified into high- and low-risk groups by utilizing the median risk score derived from this nomogram (Supplementary Figures S3A, S3B). The Kaplan–Meier survival curve analysis of the two groups demonstrated that MACE incidence was significantly greater in the high-risk group than in the low-risk group within both cohorts (P < 0.05).

## Discussion

In this study, we investigated the potential application of a predictive model based on inflammation biomarkers (mean platelet width and PLR) in the longitudinal risk assessment of patients with SCAD and intermediate coronary stenosis. Based on these four independent risk factors, we constructed a nomogram model and used multiple validations to establish the model. Our study findings showed that the predictive nomogram had acceptable discriminative performance and feasibility in predicting MACEs at 2-, 3- and 4-years in patients with SCAD and intermediate coronary stenosis. Therefore, our nomogram model is a valuable and efficient tool in the clinical management practice of SCAD with intermediate coronary stenosis.

SCAD is a complex syndrome characterized by varied treatment responses implemented according to a spectrum of clinical and imaging parameters, as well as relevant biomarkers (Rioufol et al., 2021; Kang et al., 2023; Dai et al., 2022). The phenotype characterization and prognostication in SCAD have been conventionally performed utilizing coronary angiography to assess lesion severity (Doenst et al., 2019). However, numerous studies have highlighted the substantial impact of revascularization on cardiac event rates in high-risk cohorts. Conversely, other researchers have found that the revascularization of obstructed epicardial coronary arteries in most patients with SCAD does not lead to a notable decrease in mortality or cardiovascular incidents under conditions of optimal medical therapy (Boden et al., 2026; Tang et al., 2023; Rajkumar et al., 2023). Moreover, SCAD should not be considered a static disease because its progression can be influenced by a range of factors that can potentially induce an unstable disease course. Furthermore, the prevailing research consensus advocates for an in-depth exploration of relevant therapeutic interventions, as well as transitioning from the limited emphasis on single coronary artery stenosis to a comprehensive consideration of at-risk patients (Knuuti et al., 2020). Notably, a standardized approach solely relying on coronary stenosis may be inadequate for the longitudinal management of SCAD. Currently, machine learning is transforming the analysis and interpretation of multimodal data, providing deeper insights into individual responses to coronary lesions (Ye et al., 2023; Zhou T. et al., 2024; Wang et al., 2024b; Wang et al., 2022a; Wang et al., 2022b). This not only improves diagnostic precision but also facilitates the development of personalized medicine in cardiology. Therefore, achieving a deeper understanding of the patient’s overall condition, including underlying diseases and prognostic factors linked to SCAD accompanied by intermediate coronary lesions, is critical. This insight may help enhance the disease management and outcomes for vulnerable patients, particularly those having SCAD with intermediate coronary stenosis (Song et al., 2023; Koo et al., 2022).

In our study, we initially employed the LASSO regression and boruta algorithm machine-learning approach to select features from our development cohort. The variable selection process was performed by integrating LASSO regression and the Boruta algorithm, both of which are robust methods for feature selection in high-dimensional datasets. This integration not only improves the interpretability of the model but also ensures that the selected variables are statistically significant and clinically relevant. Subsequently, a multifactorial Cox regression analysis was performed to extract independent prognostic predictors. This two-step approach enabled us to identify optimal features from high-dimensional data using machine learning techniques, while the final predictive framework relies entirely on traditional Cox regression analysis. Accordingly, we developed a nomogram that integrates inflammation biomarkers (mean platelet width and PLR) and EHR clinical data to enhance the risk stratification of patients with SCAD, particularly the sub-population having intermediate coronary lesions without PCI. Moreover, our nomogram also incorporated various patient-specific factors, such as diabetes mellitus and lipoprotein(a). This hybrid approach combines the strengths of modern machine learning for efficient feature selection with the clinical interpretability and established validation frameworks of traditional survival models (Xu et al., 2024; Wang et al., 2025). The Cox regression-based nomogram provides familiar hazard ratios and confidence intervals that facilitate clinical decision-making and regulatory acceptance in cardiovascular medicine. By integrating these diverse data points, our study provides a more holistic understanding of patient reactions to intermediate coronary lesions, which is essential for informing clinical decision-making and optimizing therapeutic strategies. Additionally, combining these inflammation biomarkers into our analytical framework facilitated a more robust characterization of cardiac pathophysiology. This integrated strategy enables clinicians to identify subtle changes in the early stages of disease through routine clinical blood tests, which are easily accessible and highly suitable for widespread clinical application. Furthermore, this enriched dataset also enhances the precision of risk stratification and allows the tailoring of therapeutic interventions for individuals with SCAD, potentially improving clinical outcomes. Moreover, this approach aligns with the current trends in personalized medicine, which essentially involve detailed phenotypic profiling for optimizing patient-specific management strategies.

Coronary artery disease (CAD) is fundamentally an inflammatory disease, characterized by chronic inflammation within the arterial walls (Henein et al., 2022; Huang and Sun, 2023; Zhang et al., 2023). This inflammatory process plays a pivotal role in the initiation, progression, and destabilization of atherosclerotic plaques (Henein et al., 2022). Biomarkers of inflammation, including NLR, PLR, and mean platelet width, are often elevated in patients with CAD, reflecting the systemic nature of this inflammatory response (Tang et al., 2023; Hu et al., 2018; Yang et al., 2020). While NLR has been widely recognized as a marker of systemic inflammation and has shown utility in acute settings, such as predicting short-term adverse events following acute coronary syndromes, its prognostic value diminishes over extended follow-up periods (Yang et al., 2020). In our study, inflammatory biomarkers, specifically mean platelet width and PLR, emerged as independent predictors of long-term MACEs in patients with SCAD and intermediate coronary stenosis. These findings underscore the critical role of systemic inflammation in the progression of CAD, even in patients with clinically stable disease. Elevated mean platelet width and PLR, reflecting a heightened inflammatory state, were significantly associated with adverse outcomes, including cardiac mortality, revascularization and myocardial infarction, over extended follow-up periods. Moreover, consistent with findings in previous studies, diabetes mellitus is a well-established and significant risk factor for coronary artery disease (Arnold et al., 2020). Consequently, diabetes management, including glycemic control and comprehensive cardiovascular risk reduction, is a critical component of strategies aimed at preventing and treating coronary artery disease in this high-risk population (Arnold et al., 2020). Additionally, lipoprotein(a), is closely linked to the inflammatory response and oxidative stress of the arterial wall, possibly affecting the progression of intermediate coronary lesions (Mehta et al., 2022). Our results highlight the importance of incorporating inflammatory-metabolic biomarkers into the prognostic evaluation of SCAD, providing valuable insights for optimizing long-term management strategies in this patient population. Thus, our model enhanced prognostic precision by integrating a constellation of Inflammation-metabolic interaction status. These study findings not only open new vistas for diagnostic and therapeutic decision-making in patients with SCAD and intermediate coronary lesions but also provide valuable insights that can advance future clinical practice and research endeavors.

### Study limitations

First, the retrospective nature of our study did not allow us to control for a few potential confounding factors, possibly causing inherent bias in the results. Hence, future prospective studies should be conducted to further validate the reliability of our model and to include more information on the effect of comorbidities and related treatments on the risk of cardiovascular disease. Second, our patient sample size was relatively small, thereby limiting the generalizability of our predictive model. Third, both our development and validation cohorts consist exclusively of Asian populations from two Chinese medical centers. Given the well-documented ethnic differences in inflammatory biomarkers (such as PLR, mean platelet width) and metabolic parameters (particularly lipoprotein(a) levels), the generalizability of our predictive model to other ethnic populations remains uncertain. The established variations in biomarker reference ranges, genetic polymorphisms affecting inflammatory responses, and population-specific cardiovascular risk profiles necessitate external validation in diverse ethnic cohorts before widespread clinical implementation. Finally, it is necessary to integrate more types of cardiac imaging data, especially cardiac CT or coronary angiography data, to better assess the characteristics of high-risk plaques and the overall burden of atherosclerosis, and to further improve our risk assessment models.

## Conclusion

In this study, we developed a prognostic nomogram model utilizing inflammation biomarkers (mean platelet width and PLR) and EHR data that provided individualized risk stratification for MACEs at 2-, 3- and 4-year horizons in patients with SCAD and intermediate coronary lesions. Our model, which has the potential to advance through longitudinal patient tracking and data analytics, aims to become an indispensable tool in the clinicians’ armamentarium for assessing patient risk and formulating personalized treatment strategies. Furthermore, this predictive model will contribute to optimizing therapeutic outcomes and minimizing unwarranted medical interventions in this specific patient population.

## Data Availability

The original contributions presented in the study are included in the article/Supplementary Material, further inquiries can be directed to the corresponding authors.
