# Supplementary material for: Machine learning-selected inflammation biomarkers for stable coronary artery disease with intermediate coronary lesions: potential for long-term prognosis in a multicenter cohort study
Source: Front Physiol. 2026 Feb 17;17:1688153. doi: 10.3389/fphys.2026.1688153 (PMC12953134; doi:10.3389/fphys.2026.1688153)

**Supplemental Figure 1. The time-AUC curves of the prediction model display the time-dependent AUC values for development (A) and validation (B) cohorts across various time points.**


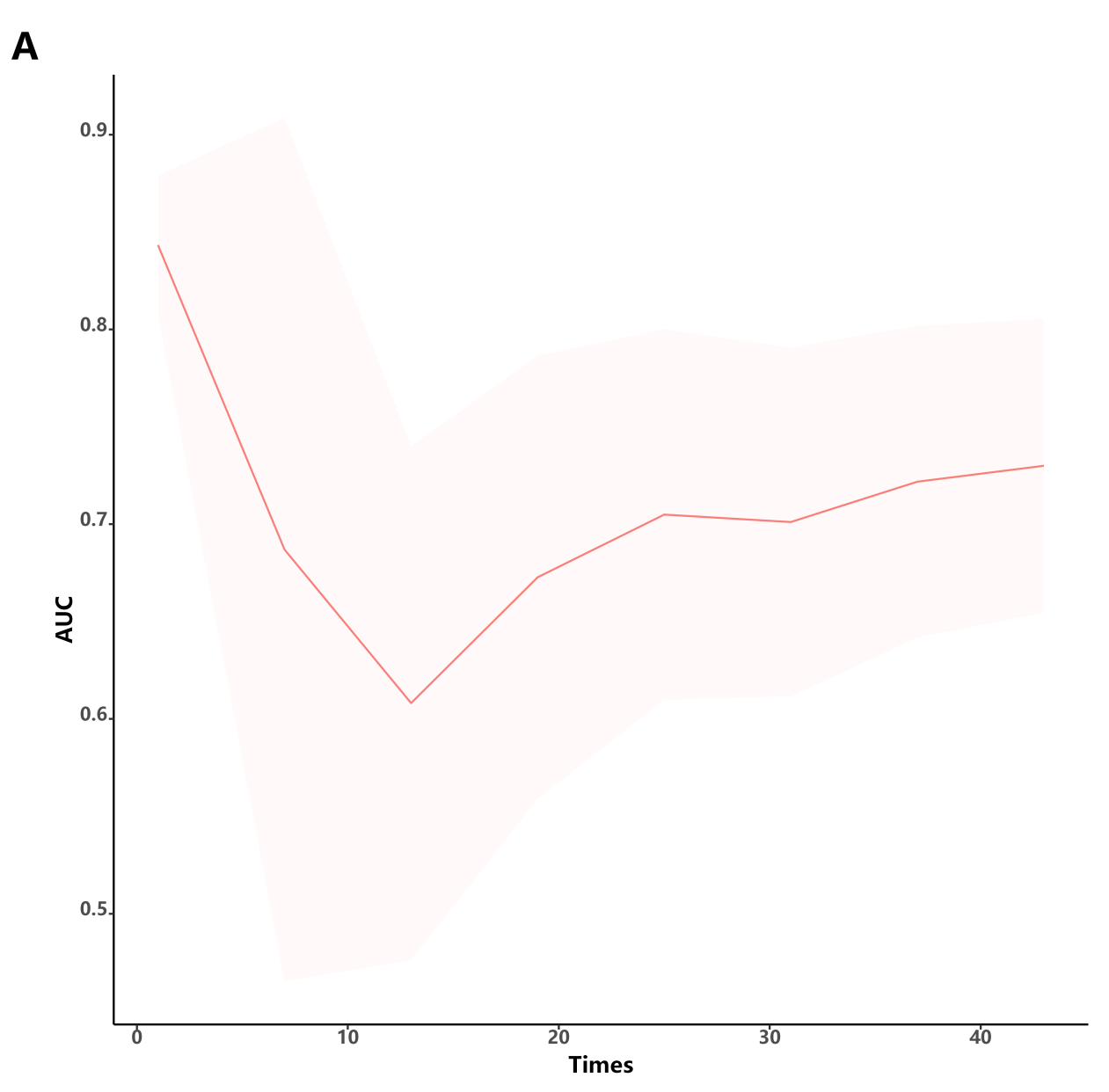

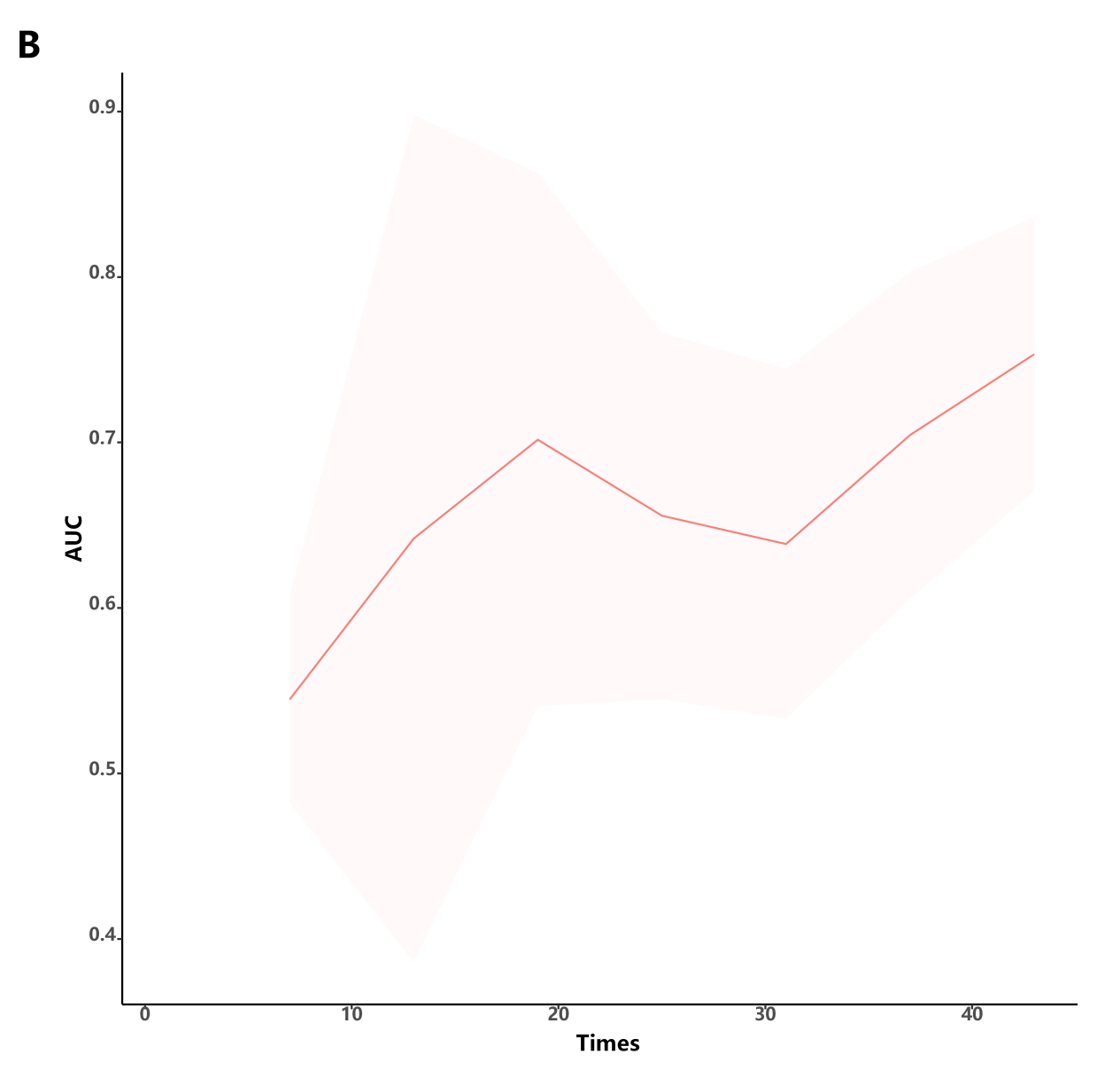


**Supplemental Figure 2. Decision curve analysis (DCA) analysis of the predictive nomogram and the five univariate individual models in the development (A C and E) and validation cohorts (B, D and F), respectively.**


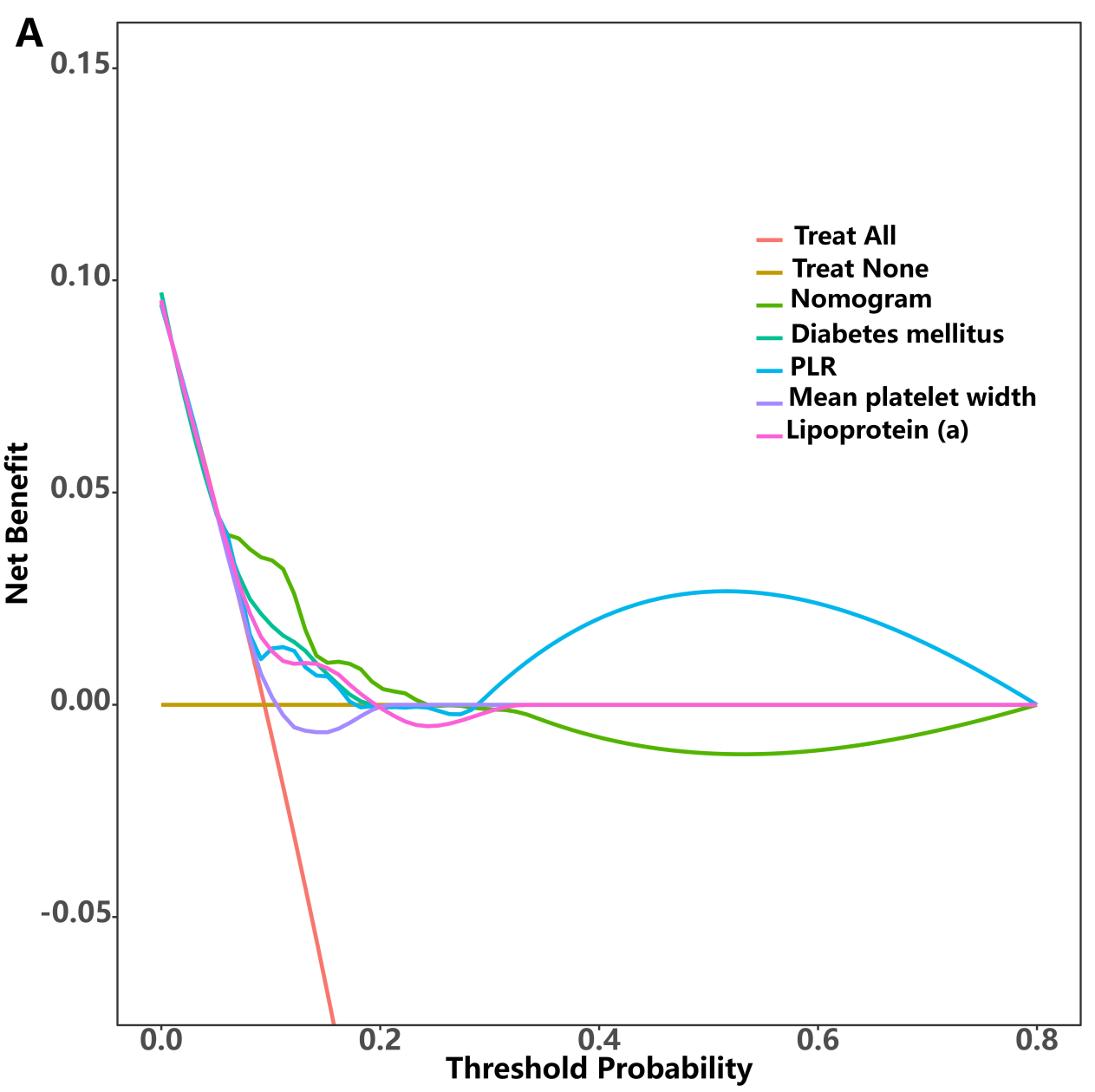

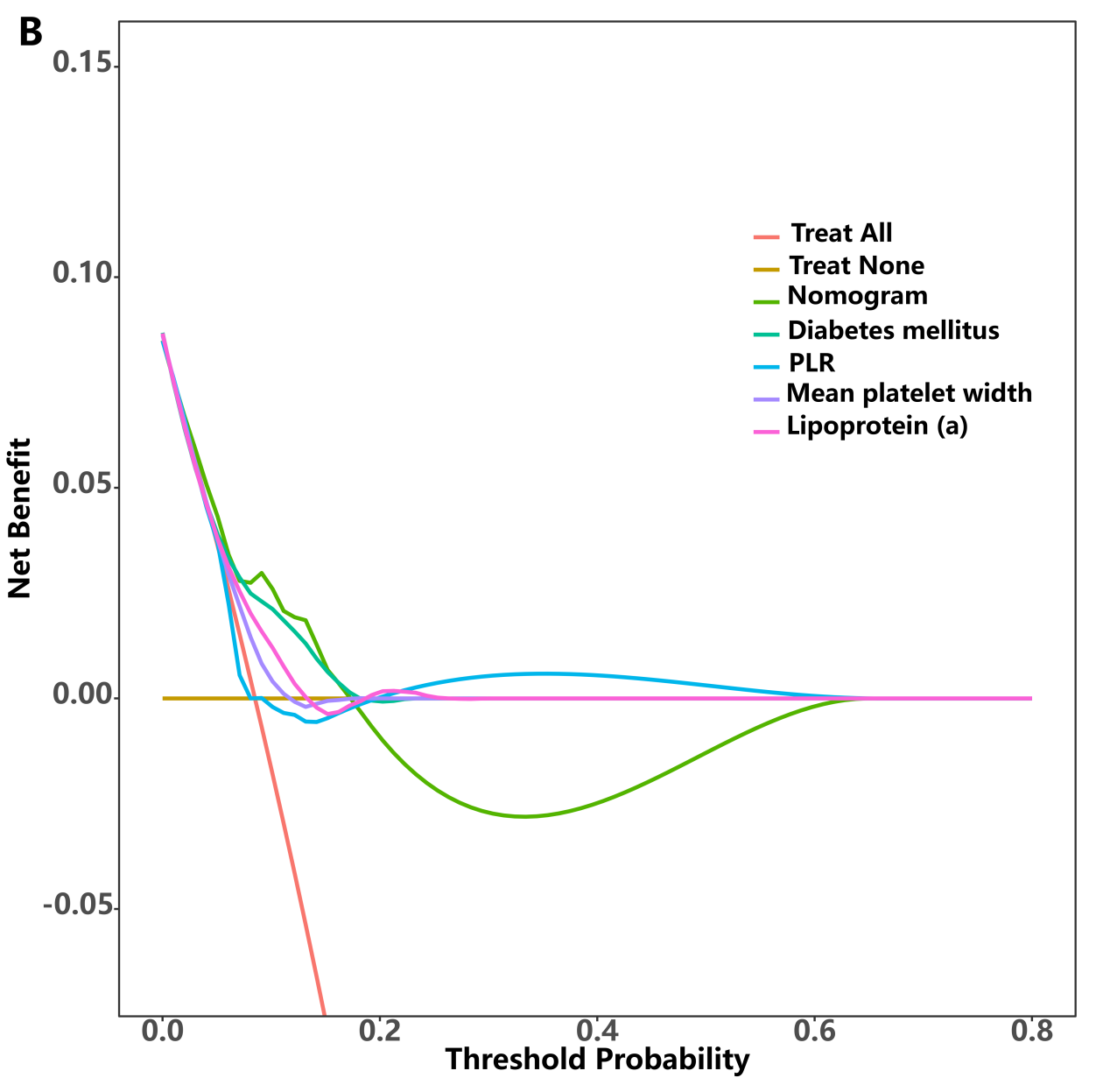

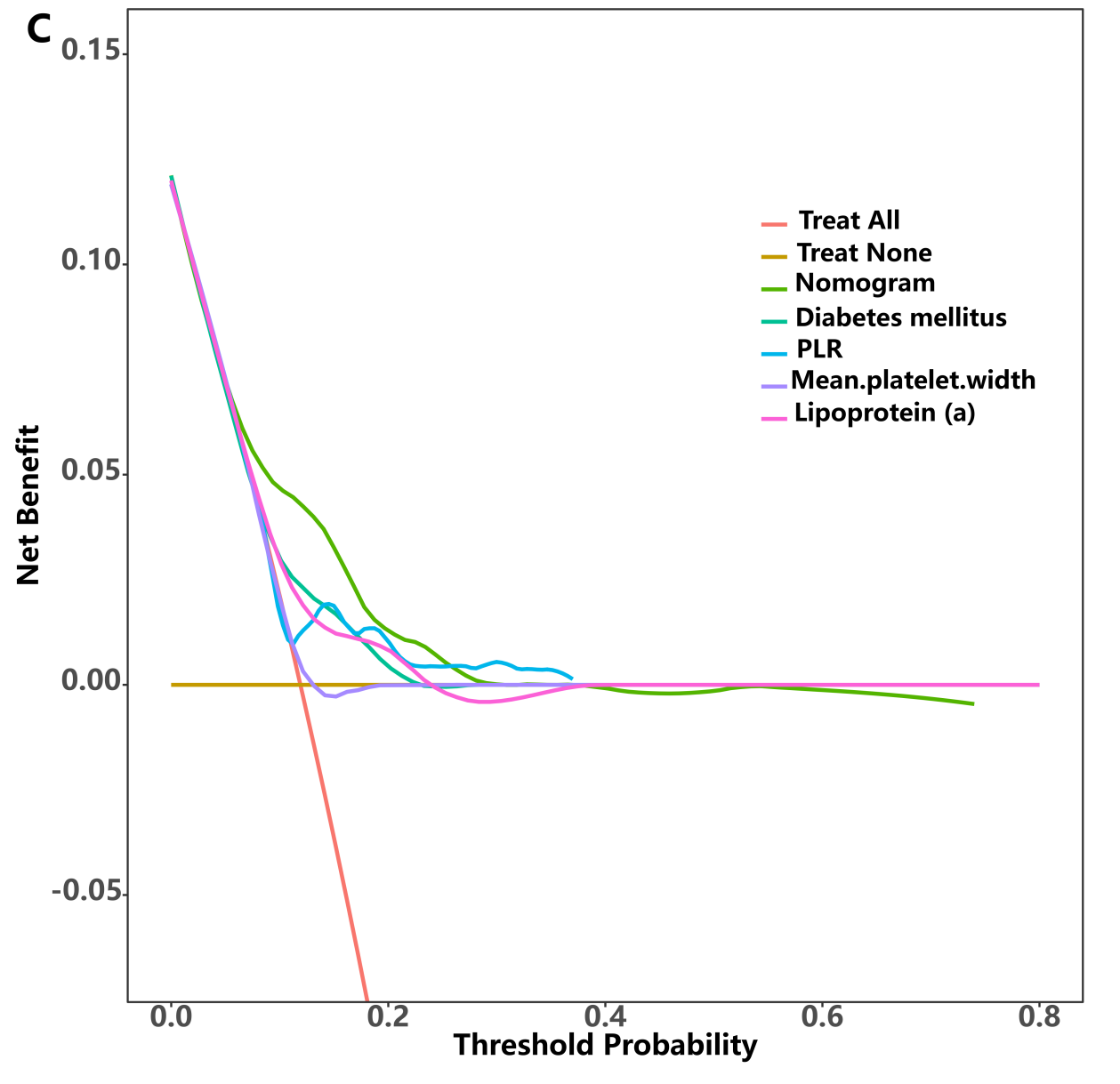

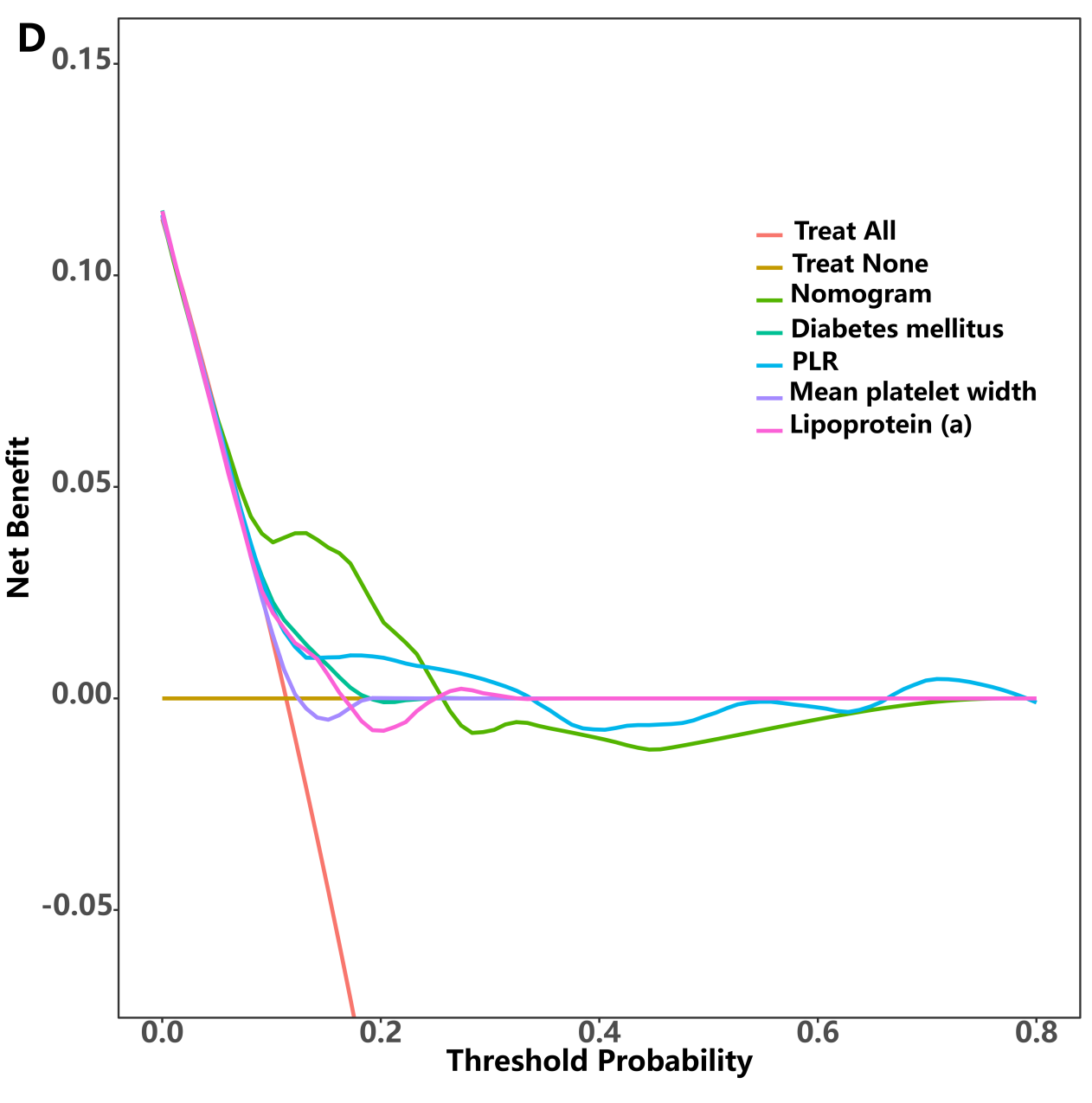

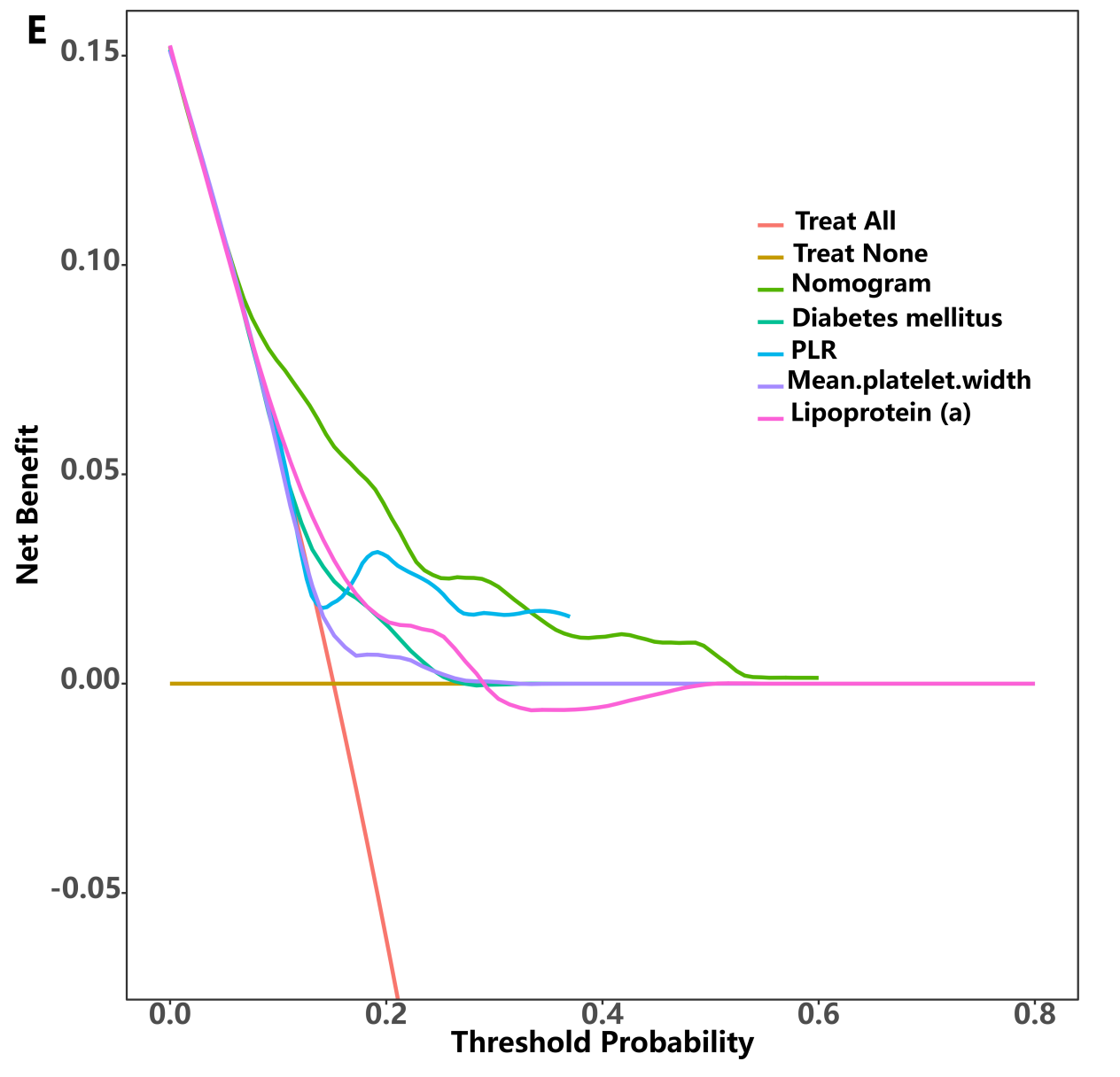


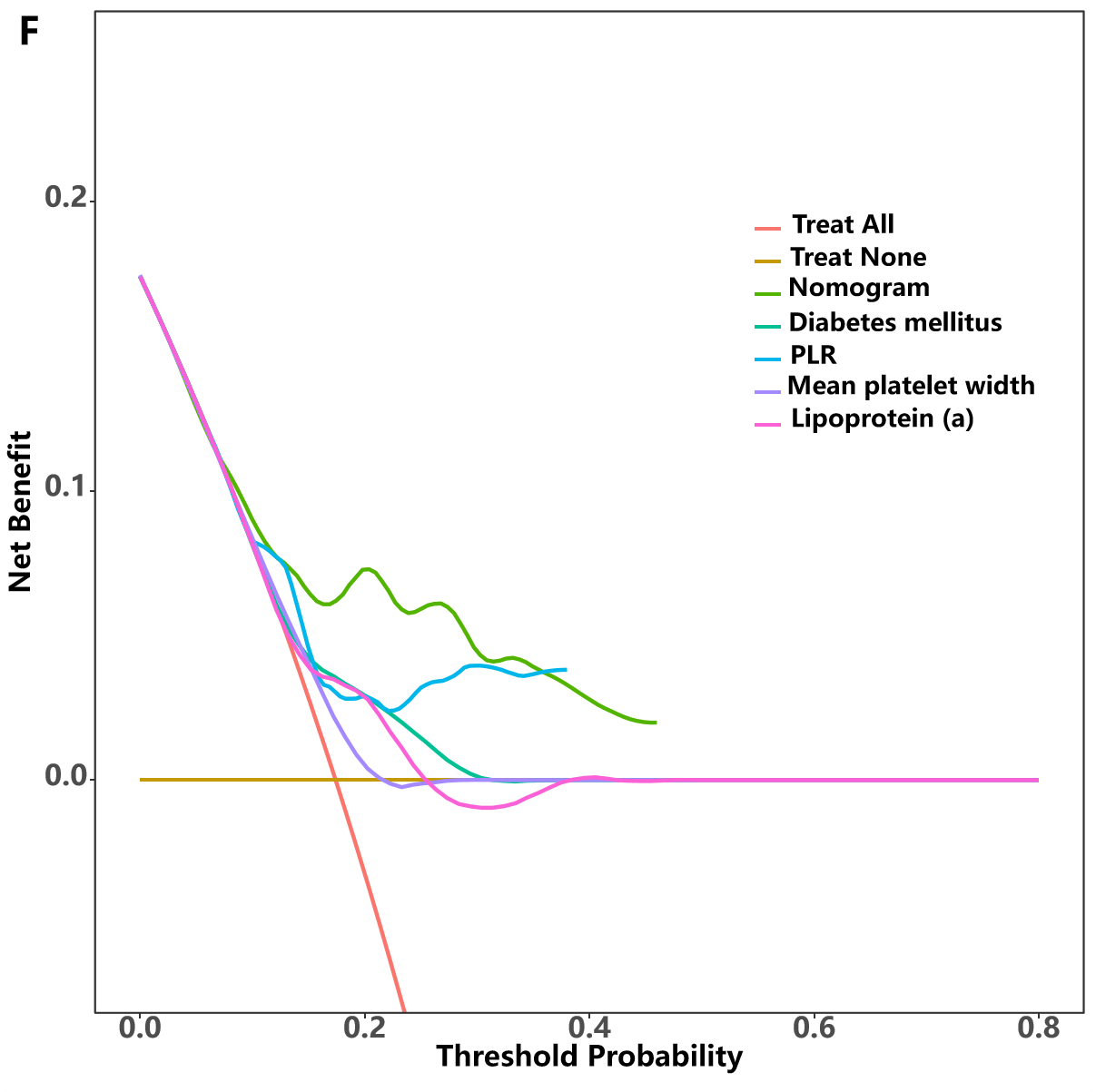


**Supplemental Figure 3. The cumulative survival without MACEs was analyzed in patients categorized as high risk or low risk based on the nomogram score. This analysis was performed separately for both the development cohort (A) and validation cohort (B).**


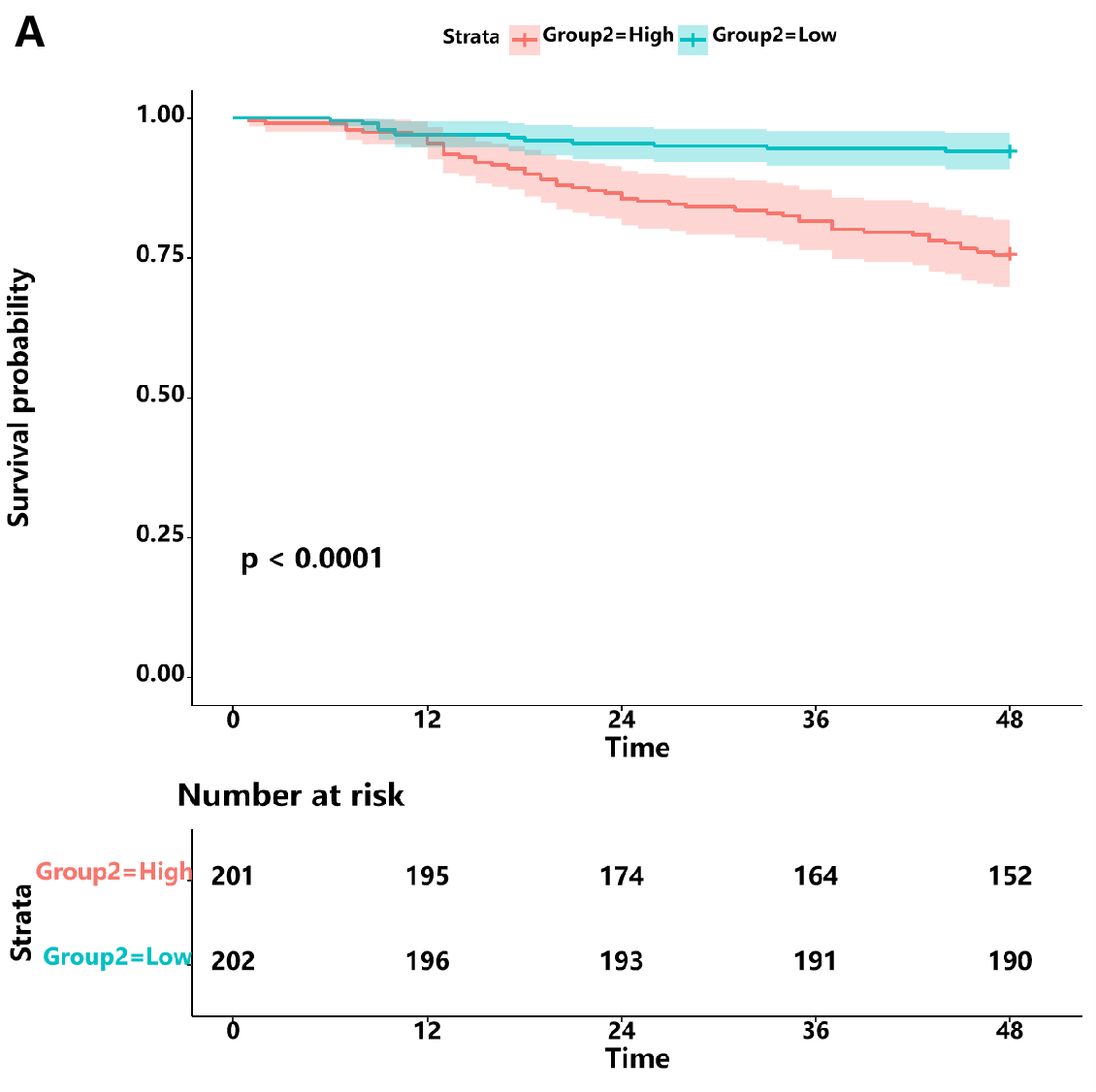


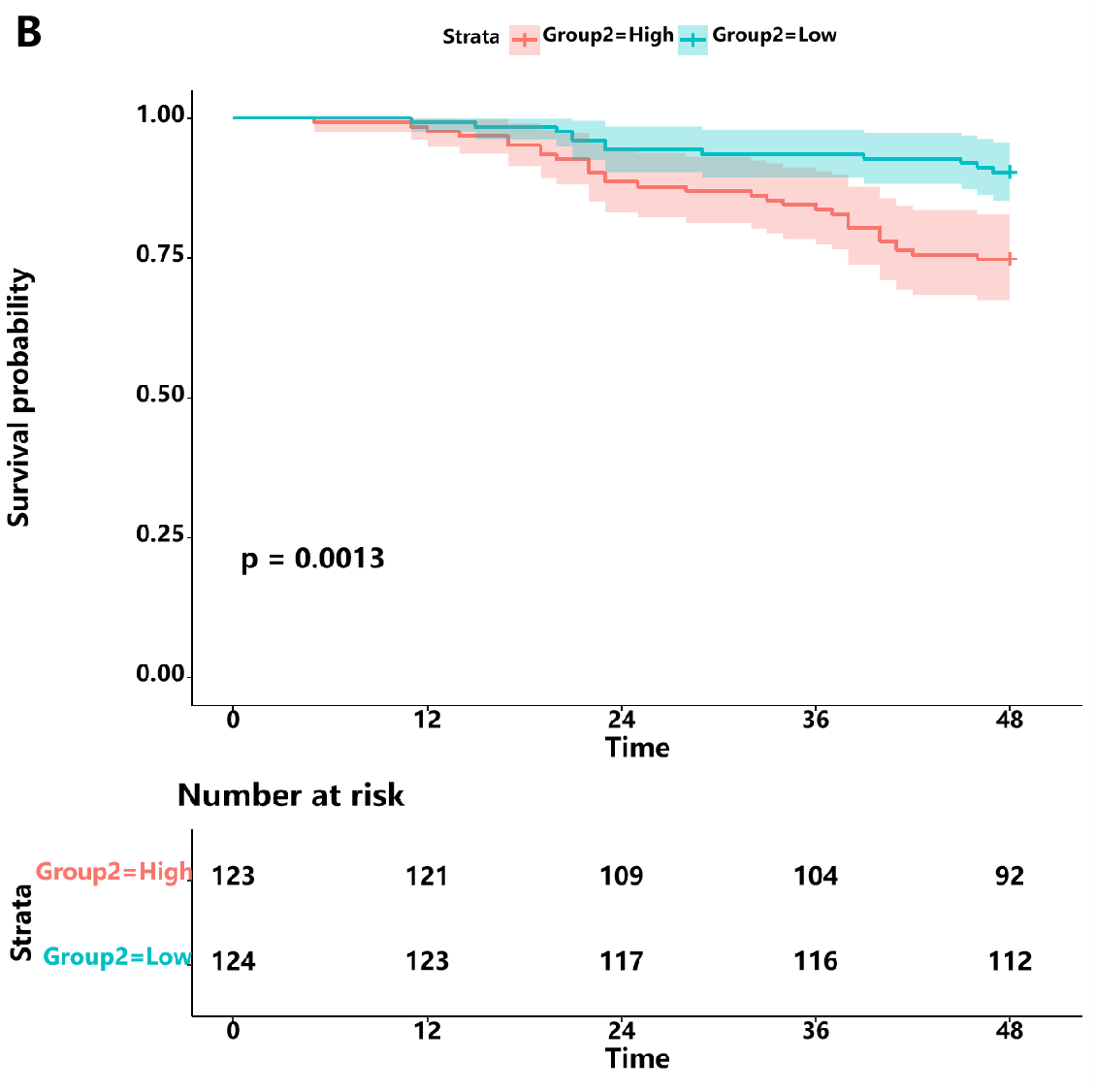

Supplement: Supplementary file 1 [file DataSheet1.docx]
